# The Microscopic Anatomy of the Human Body in Health and Disease

**Published:** 1847-01

**Authors:** 


ART. XIX.—*The Microscopic Anatomy of the Human Body in Health and Disease, illustrated with numerous Drawings in Colour.* By ARTHUR HILL HASSALL, F.L.S. M.R.C.S., &c. Parts III-V. 8vo, pp. 114. With ten Plates.

WE should not have so soon returned to this publication, but for the sake of redeeming a pledge which we gave in our former notice of it, to speak more favorably of its execution, should the improved character of the work as it proceeded, enable us to do so. It is now our pleasing duty to state, that there is not only a considerable amendment in the plates of the three numbers before us, but that in the fifth part are given seven additional plates, to replace those issued in the early numbers, to which they are vastly superior, both in drawing and colouring. The justice of that part of our criticism which relates to the plates has thus been practically admitted by the author and his artist. It is right that we should further mention that the proportion of figures taken from the human subject is much increased in the later numbers. But we must still express our conviction that the work cannot be comprised within anything like the limits first announced, without doing injustice to a large proportion of the topics it is intended to embrace. For in the five numbers now issued, the animal *fluids* (blood, mucus, pus, milk, and semen) are not completed; and less than seven parts remain, therefore, for the description of all the *solid* textures of the human body. Moreover, of the large amount of letter-press which accompanies the plates, the greater part is occupied by details of a chemical nature, altogether foreign to the subject. The author seems to forget that he is writing about *Microscopic* Anatomy, not *General* Anatomy; and the investigations of Andral and Gavarret, Becquerel and Rodier, &c., on the proportion of the different constituents of the blood, have not a more just place in it than a full discussion of the microscopic characters of the blood would have in Turner's Chemistry. We are still constrained to say that Mr. Hassall appears to have entered upon his work too hastily; and that he seems ignorant of much that is current amongst intelligent microscopists at the present time, his acquaintance with the subject lying too much in the past.

